# Optimizing Ethanol Production in *Saccharomyces cerevisiae* at Ambient and Elevated Temperatures through Machine Learning-Guided Combinatorial Promoter Modifications

**DOI:** 10.1021/acssynbio.3c00199

**Published:** 2023-09-08

**Authors:** Peerapat Khamwachirapithak, Kittapong Sae-Tang, Wuttichai Mhuantong, Sutipa Tanapongpipat, Xin-Qing Zhao, Chen-Guang Liu, Dong-Qing Wei, Verawat Champreda, Weerawat Runguphan

**Affiliations:** † National Center for Genetic Engineering and Biotechnology (BIOTEC), National Science and Technology Development Agency (NSTDA) 111 Thailand Science Park, Phahonyothin Road, Khlong Nueng, Khlong Luang, Pathum Thani 12120, Thailand; ‡ State Key Laboratory of Microbial Metabolism, Joint International Research Laboratory of Metabolic & Developmental Sciences, School of Life Sciences and Biotechnology, Shanghai Jiao Tong University, Shanghai 200240, People’s Republic of China; § Department of Bioinformatics and Biological Statistics, School of Life Sciences and Biotechnology, Shanghai Jiao Tong University, Shanghai 200240, People’s Republic of China

**Keywords:** machine learning, ethanol, yeast, stress tolerance, ensemble decision tree

## Abstract

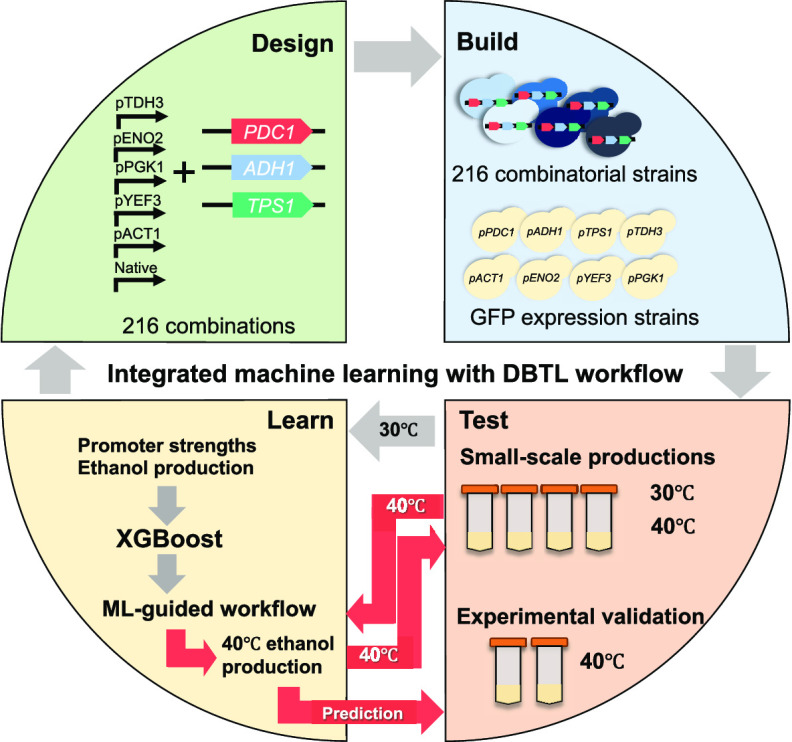

Bioethanol has gained popularity in recent decades as an ecofriendly alternative to fossil fuels due to increasing concerns about global climate change. However, economically viable ethanol fermentation remains a challenge. High-temperature fermentation can reduce production costs, but *Saccharomyces cerevisiae* yeast strains normally ferment poorly under high temperatures. In this study, we present a machine learning (ML) approach to optimize bioethanol production in *S. cerevisiae* by fine-tuning the promoter activities of three endogenous genes. We created 216 combinatorial strains of *S. cerevisiae* by replacing native promoters with five promoters of varying strengths to regulate ethanol production. Promoter replacement resulted in a 63% improvement in ethanol production at 30 °C. We created an ML-guided workflow by utilizing XGBoost to train high-performance models based on promoter strengths and cellular metabolite concentrations obtained from ethanol production of 216 combinatorial strains at 30 °C. This strategy was then applied to optimize ethanol production at 40 °C, where we selected 31 strains for experimental fermentation. This reduced experimental load led to a 7.4% increase in ethanol production in the second round of the ML-guided workflow. Our study offers a comprehensive library of promoter strength modifications for key ethanol production enzymes, showcasing how machine learning can guide yeast strain optimization and make bioethanol production more cost-effective and efficient. Furthermore, we demonstrate that metabolic engineering processes can be accelerated and optimized through this approach.

## Introduction

The growing demand for bioethanol production stems from its benefits over fossil fuels such as carbon neutrality and reduced environmental impact. As a favored biofuel and valuable plant biomass byproduct, ethanol is extensively utilized in vehicles and engines on its own or mixed with gasoline. *Saccharomyces cerevisiae* is recognized as the most efficient bioethanol producer.^1^ Several studies have been conducted to improve ethanol production by yeast. However, traditional methods for the metabolic engineering of biofuel production are expensive and time-consuming, often involving many iterations of similar experiments. Directed evolution, a more recent technique, exposes yeast to specific stressors over multiple culture rounds, yielding evolved strains with the desired traits. However, this method is not always effective, especially when the desired trait is not linked to growth. Heterologous pathway engineering, another approach, relies on prior knowledge of the pathway of interest and may not consistently deliver results.^2^

Previous research has pinpointed the enzymes pyruvate decarboxylase Pdc1p (encoded by *PDC1*) and alcohol dehydrogenase Adh1p (encoded by *ADH1*) as key factors in yeast ethanol production.^3−6^ Pdc1p catalyzes the decarboxylation of pyruvate to acetaldehyde, while Adh1p reduces acetaldehyde to ethanol. Overexpression of *PDC1* and *ADH1* results in a 1.4-fold increase in ethanol production in modified *S. cerevisiae* strains using glycerol as a substrate.^7^ A similar enhancement in ethanol production was observed in another yeast strain, *Hansenulapolymorpha*.^8^ However, the overexpression of *PDC1* alone does not boost ethanol production.^9^ Consequently, *PDC1* and *ADH1* are vital for ethanol production and represent potential targets for optimization through expression tuning.

During ethanol production, high ethanol concentrations can induce stress, inhibiting yeast growth and eventually causing cell death.^10^ One strategy to increase ethanol production is to reduce glycerol production, a byproduct, although glycerol can enhance tolerance to specific stresses.^11,12^ Heat stress is another critical factor during ethanol production that can inhibit the growth and fermentation efficiency. This increases the cost of cooling processes needed to maintain optimal fermentation temperatures of 30 °C.^13−15^ In the context of yeast, high-temperature stress disrupts membrane fluidity and protein structure and function.^16,17^ To counter heat stress, researchers have developed thermotolerant yeast strains through adaptive evolution and metabolic engineering. One study reported that enhanced ergosterol biosynthesis improved growth and ethanol production in adaptively selected yeast.^18^ Another study discovered that trehalose accumulation in response to ethanol stress increased ethanol production by approx. 2-fold at 38 °C when *TPS1* was overexpressed.^19^ The overexpressed *TPS1* transformant also demonstrated improved growth at temperatures up to 42 °C.^20^

Despite their widespread use in academia and industry, directed evolution and metabolic engineering approaches are often costly and time-consuming, necessitating multiple experimental iterations. Recently, machine learning (ML) has emerged as a promising complement to metabolic engineering, utilizing algorithms and computational power to predict or classify output data.^21−26^ For instance, a multilayer perceptron was trained on mRNA levels of key genes involved in lycopene production in bacteria, and the ML model predicted overexpressed genes that led to an 8-fold increase in lycopene production^27^ and in protein production optimization.^28^ In another study, a combination of support vector machine, gradient-boosted trees, and neural networks demonstrated high accuracy in predicting output in an *Escherichia coli* factory^29^ and in monoterpenoid optimization using support vector regression.^26^ ML was applied to the design–build–test–learn (DBTL) cycle to optimize dodecanol production in *E. coli*.^30^ Similarly, in yeast, an ML workflow integrated the optimization of the heterologous pathway assembly and successfully improved violacein and β-carotene production using an artificial neural network (ANN)^31^ and increased tryptophan production.^32^ These findings suggest that ML has significant potential to expedite and optimize metabolic engineering processes.

In this study, we aim to create an ML workflow capable of accelerating the optimization of ethanol production in *S. cerevisiae* at both ambient (30 °C) and elevated temperatures (40 °C). This ML workflow was constructed based on data derived from ethanol production at a temperature of 30 °C. To enhance ethanol production, we substituted the native promoters of the *ADH1*, *PDC1*, and *TPS1* genes with five promoters of varying strengths. This process resulted in the generation of 216 combinatorial strains of *S. cerevisiae*, including the wild-type strain. The metabolites and promoter strengths gleaned from the 30 °C experiment facilitated the construction of the ML-guided workflow. We used the XGBoost algorithm for intensive hyperparameter tuning, which yielded a model with higher performance than linear regression, generalized linear regression, decision tree, random forest, and support vector machine. We then applied this ML workflow to predict ethanol production at 40 °C by sampling 31 combinatorial yeast strains with various combinations of promoter strengths and metabolite levels, leading to the identification of improved strains in the second round of ML prediction. Our study provides a valuable combinatorial library of promoter strength modifications for essential enzymes involved in ethanol production, highlighting the power of machine learning in guiding strain optimization in yeast under varying temperature conditions.

## Results and Discussion

### Combinatorial Library Construction and Overall ML-Guided DBTL Workflow

**Figure 1:**
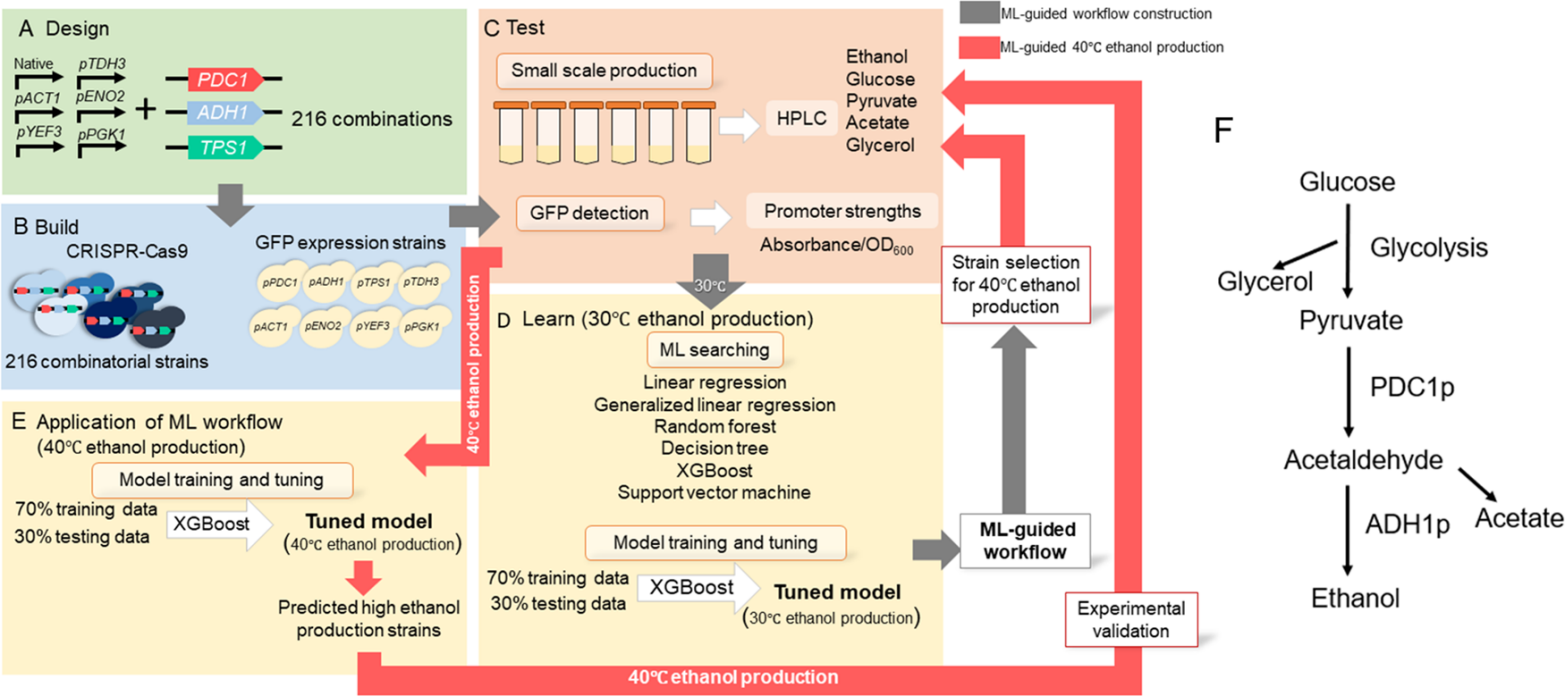
Integration of machine learning into the DBTL workflow for ethanol optimization. (A) Design: five promoters from yeast were used to replace the native promoters of *PDC1*, *ADH1*, and *TPS1* genes, which were combined into 216 combinations to construct the combinatorial library. (B) Build: the CRISPR/Cas9 system was applied to replace the native promoter in the WT yeast, using *URA3* as a marker. To determine promoter strengths, promoters were placed upstream of the *GFP* gene and transformed into wild-type yeast. (C) Test: small-scale ethanol production from 216 combinatorial strains was performed. Ethanol and other metabolites were quantified and used as an input for machine learning (ML). GFP intensity was detected using an ultraviolet–visible (UV–vis) spectrophotometer and normalized by OD_600_ to determine the promoter strength. All experiments were performed with three biological replicates. (D) Learn: ML algorithms that exhibited high performance using promoter strength, ethanol, and other metabolites as input and predicted output were selected to intensively train the ML model. Data were divided into 70% training and 30% test data. After the ML workflow was constructed, 31 strains were selected for ethanol production at 40 °C. All parameters were measured and quantified in the same way as in the 30 °C experiment. (E) Application of the ML workflow: data from the 40 °C experiment were used to train the new ML model and predict the top candidate strain for experimental validation. (F) The ethanol pathway starts from glucose as a carbon source. The Pdc1p enzyme converts pyruvate from glycolysis to acetaldehyde, which is then converted to ethanol by the Adh1p enzyme. On the other hand, glycerol can be generated from an intermediate metabolite of glycolysis, and acetate can be converted from acetaldehyde, which is also an intermediate to ethanol.

To optimize ethanol production by fine-tuning the expression of *PDC1*, *ADH1*, and *TPS1*, we replaced their native promoters with five yeast promoters including *pTDH3*, *pENO2*, *pPGK1*, *pACT1*, and *pYEF3*. Pyruvate, the final product of glycolysis, is converted to acetaldehyde by pyruvate decarboxylase (Pdc1p), and acetaldehyde is then reduced to ethanol by alcohol dehydrogenase (Adh1p). These two enzymes play a major role in yeast ethanol production, while trehalose accumulation may enhance heat stress tolerance by modifying *TPS1* expression (Figure 1F). Using the CRISPR–Cas9 system, we constructed 216 combinatorial strains (including the wild type (WT)) by replacing the native promoters of *PDC1*, *ADH1*, and *TPS1* (Figure 1A,B). We transformed CRISPR–Cas9 and guide RNA plasmids, along with the promoter donor DNA, into WT yeast. The CRISPR–Cas9 guide RNA complex then creates a cut at the specific promoter target. Homologous recombination between the native promoter and the donor DNA then replaces the native promoter sequences of target genes with new promoter sequences. The combinatorial strains were named according to the change of promoter, following the order of *PDC1*, *ADH1*, and *TPS1*. For example, the TAx strain represented the yeast that contained the *pTDH3* promoter at *PDC1*, the *pACT1* promoter at *ADH1*, and the native promoter of *TPS1* (details in the Materials and Methods section and Supporting Figure 1).

Next, we performed ethanol production on a small scale of 5 mL of YPD10 medium using all 216 combinatorial strains with three biological replicates. We quantified ethanol and other cellular metabolites, including acetate, pyruvate, and glycerol, as well as the remaining glucose in the media by HPLC. To measure promoter activities, we transformed plasmids containing promoter sequences of *pTDH3*, *pENO2*, *pPGK1*, *pACT1*, *pYEF3*, *pPDC1*, *pADH1*, and *pTPS1*, placed upstream of the *GFP* gene, into the WT yeast. We detected GFP intensity, which was then used to quantify the promoter activities and was used in ML analysis. These processes are referred to as the “Design”, “Build”, and “Test” in the DBTL cycle.

In the “Learn” phase, we implemented a machine learning model to learn from promoter strength combinations that led to high ethanol production. To find suitable ML algorithms, we first explored six ML algorithms using the caret package^33^ (Figure 1D). We then selected XGBoost for model training and tuning using 70% of the data and finally evaluated the tuned XGBoost model with the remaining 30% of the data to demonstrate the performance of the ML-guided workflow. We applied the same ML-guided workflow to optimize ethanol production under heat stress conditions (Figure 1E), selecting 31 strains from 216 combinatorial strains and performing ethanol fermentation at 40 °C. Ethanol and other metabolites were quantified using HPLC, as in the previous experiment. We also determined promoter strength under heat stress conditions for ML prediction (Figure 1D,1C). Lastly, we validated that the 10 predicted strains showed improvement in the second round of ethanol optimization under heat stress conditions (Figure 1E,1C).

### Promoter Strength of *pPDC1* Has Greater Influence on Ethanol Production at 30 °C than *pADH1*

**Figure 2:**
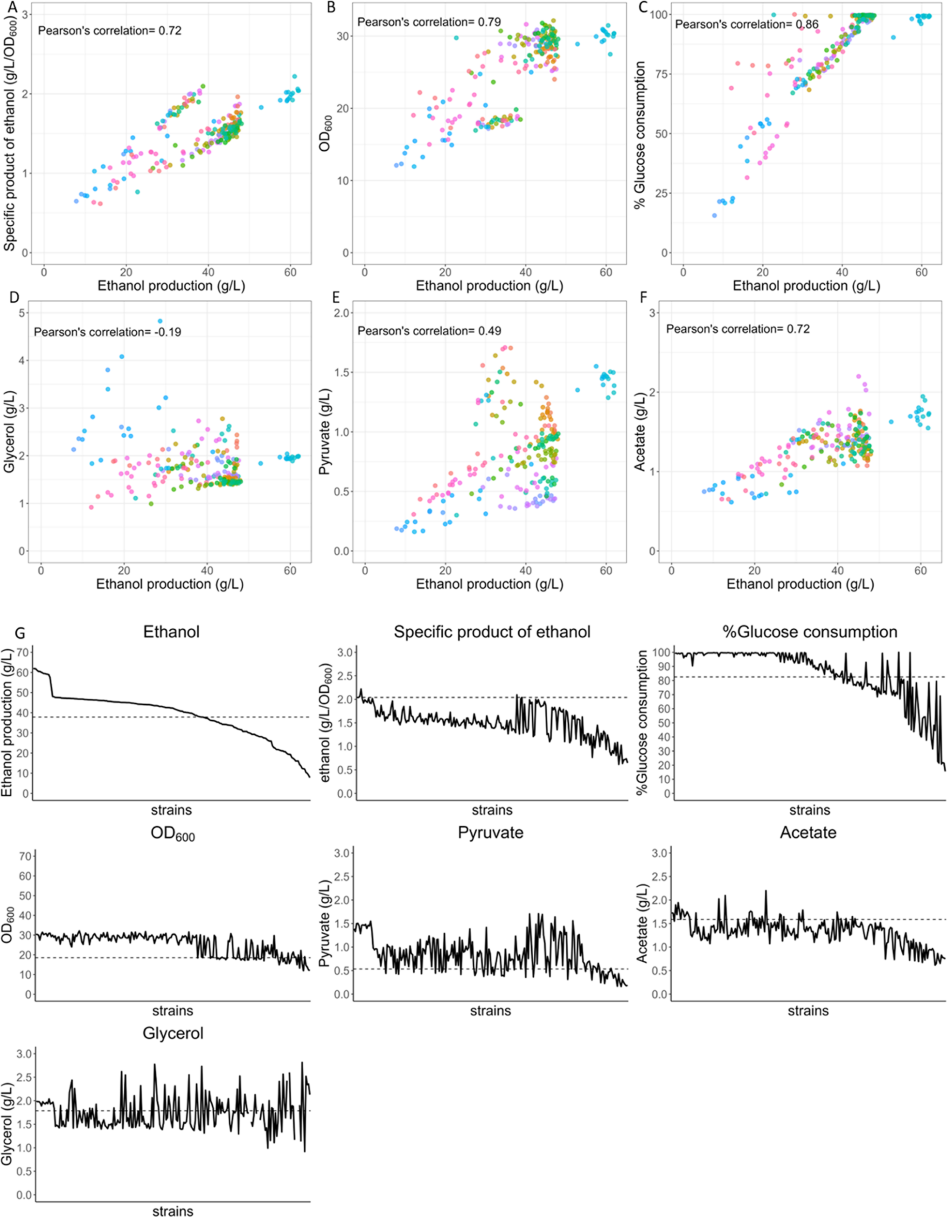
Relationship between ethanol production and production of other metabolites of all 216 combinatorial strains at 30 °C. Ethanol production of all 216 combinatorial strains was plotted against (A) specific ethanol production, (B) OD_600_, (C) % glucose consumption, (D) glycerol production, (E) pyruvate concentration, and (F) acetate concentration. Pearson’s correlations between the total ethanol production and specific ethanol production, OD_600_, % glucose consumption, and acetate concentration were higher than 0.72, while there was a slight anticorrelation (−0.19) with glycerol production. Strain annotation is in Supporting Figure 1. (G) The 216 strains (*x*-axis) were ordered according to the level of ethanol production, from high to low, and then applied to other parameters to observe the relationship of each parameter. The horizontal dashed line represents the baseline from the wild type (WT).

To investigate ethanol production from combinatorial strains, we conducted small-scale ethanol fermentations using all 216 combinatorial strains at an optimal growth temperature of 30 °C. Using WT yeast as the baseline control, we observed that 131 strains (60.65%) showed higher ethanol production titer than the WT (37.83 ± 4.41 g/L). Interestingly, among the 15 highest ethanol-producing strains, the *PDC1* gene was predominantly driven by *pTDH3*. In contrast, the *ADH1* gene was mainly driven by *pACT1*, *pPGK1*, and *pENO2*. *TPS1*’s promoter was *pYEF3*, *pACT1*, *pENO2*, *pPGK1*, or *pTHD3*. Ethanol production from these combinations varied between 52.87 ± 8.15 and 61.96 ± 0.97 g/L, signifying a substantial increase of approx. 39–63% over the wild type. These strains are represented as shades of blue, particularly in the top-right portion of each panel in Figure 2A–F (refer to Supporting Figure 1 for strain identification). Furthermore, we found that a single promoter change also improved ethanol production compared to the WT strain (e.g., xxK, xxA, xxN, xxY, xxT, xYx, and Kxx; see Supporting File 1). All improvements were attributed to increases in promoter strength from their native promoters. The *pTDH3* has been reported to have relatively high promoter activity among our selected promoters, while *pACT1*, *pPGK1*, and *pENO2* activities were lower.^34^ Our findings suggest that a high promoter activity of *PDC1*, in combination with a moderate promoter activity of *ADH1*, is necessary to significantly enhance ethanol production. This is consistent with studies showing a 1.4-fold increase in ethanol production from glycerol upon the overexpression of *PDC1* and *ADH1* using promoters of equal strengths.^7^ In the yeast *H. polymorpha*, the overexpression of *PDC1* and *ADH1* led to an impressive 5.4-fold and 3.3-fold increase in ethanol conversion from glycerol, respectively.^8,35^ However, it is essential to note that the ethanol levels in the wild-type (WT) strain in these earlier studies were significantly lower, exhibiting 50–100 times less ethanol production compared to the WT strain in our study. This discrepancy can likely be attributed to the nonconventional strains and media used in the earlier studies. Our results underscore that fine-tuning the promoter strengths of key enzymes in the ethanol pathway can notably enhance ethanol production in *S. cerevisiae*, a conventional and industrially important yeast species.

We calculated the correlations of ethanol production with the production of other intermediate metabolites, such as glycerol, pyruvate, and acetate, as well as parameters that indicated yeast growth, including glucose consumption and OD_600_. The aim was to determine the effect of changes in ethanol production on metabolic fluctuations. High ethanol-producing strains can be distinguished by high-specific ethanol production, high glucose consumption, and growth, as measured by OD_600_. Although ethanol production, specific ethanol production, OD_600_, and glucose consumption were highly correlated, some strains with high OD_600_, specific ethanol production, and glucose consumption did not produce as much ethanol as others (Figure 2A–C).

When considering the relationship between ethanol production and the production of intermediate metabolites, we found that ethanol had a high correlation with acetate (Pearson’s correlation of 0.72), while pyruvate was moderately associated with ethanol (Figure 2E,F). High levels of pyruvate, acetate, and glycerol, along with high growth and glucose consumption, were found in the top 15 strains of ethanol production mentioned earlier. However, these characterizations were not sufficient to identify other high ethanol producers. Glucose consumption showed a proportional change with ethanol production (Figure 2G), indicating that consumed glucose is directly related to ethanol production. Conversely, changes in measured pyruvate, acetate, and glycerol, which are intermediate metabolites, did not have a linear relationship with ethanol production.

### Decision Tree-Based Machine Learning Performs Best in Predicting Ethanol Production

**Figure 3:**
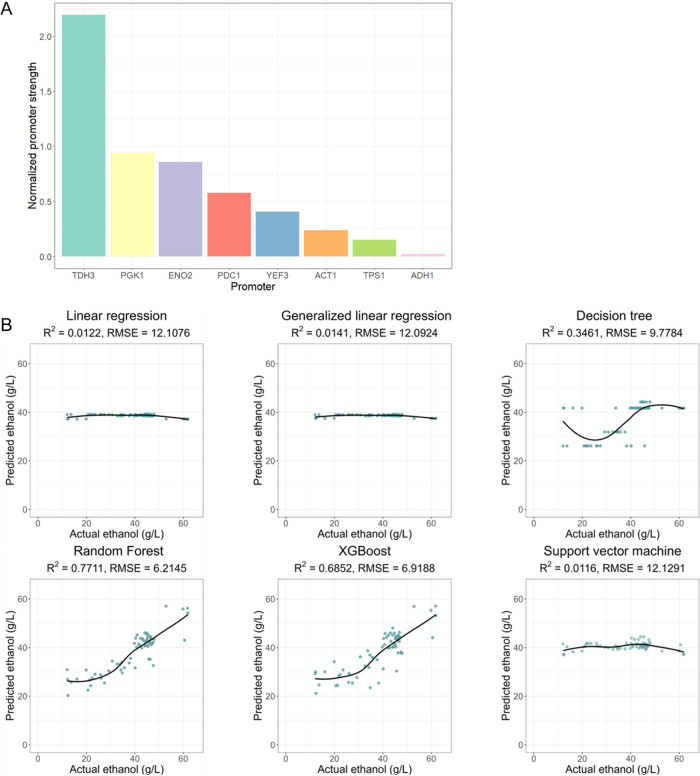
Decision tree-based algorithms demonstrate high model performances. (A) GFP intensities were normalized by OD_600_ and then scaled for use as input in the ML training of ethanol production at 30 °C. (B) ML performance from six ML algorithms, including linear regression, generalized linear regression, decision tree, random forest, XGboost, and support vector machine, was evaluated using test data (64 strains, 30%) to determine the *R*-square of each model. The RMSE was calculated from training models using training data (70%, 152 strains). Decision tree, random forest, and XGboost exhibited high *R*-square values, representing algorithms that utilized decision tree-based approaches.

To optimize ethanol production using machine learning, we integrated promoter strengths calculated from the green fluorescence intensity of yeast containing GFPuv plasmids. The fluorescence intensity was normalized by OD_600_. We subtracted the background plasmid intensity from all promoter strengths and scaled them as input for ML training (Figure 3A). Consistent with a previous report,^36^*pTDH3* was the strongest promoter. *pPGK1*, *ENO2*, and *pPDC1* showed moderate promoter activities, while *pADH1* was the weakest promoter. We applied a flexible machine learning library called caret to search for ML algorithms that exhibit high accuracy and low error in predicting ethanol production. We used relative scaling of promoter strengths as the input and ethanol production as the predicted output and tested six machine learning algorithms, including linear regression, generalized linear regression, decision tree, random forest, XGboost, and support vector machine, with default parameters and settings to train the model. Data were randomly divided into a 70% training set and a 30% test set. We used *R*-square (*R*^2^), mean absolute error (MAE), and root-mean-square error (RMSE) to determine the accuracy and error of the training model performances. Linear regression and support vector machine regression exhibited near-zero *R*-square values and high training model errors compared with the other methods. Random forest and XGboost showed the highest *R*-square values for the testing at 0.77 and 0.69, respectively, while the *R*-square value for the decision tree model was 0.34. This suggested that methods using the decision tree process as a principle algorithm, such as the decision tree, random forest, and XGboost, were more suitable for predicting ethanol production based on promoter strength alterations (Figure 3B and Supporting Table 1). We then selected the XGboost algorithm using the XGBoost package for intensive machine learning analysis because it is more effective and flexible in model tuning and regularization.^37,38^

### XGBoost Hyperparameter Tuning and Prediction of Ethanol Production

**Figure 4:**
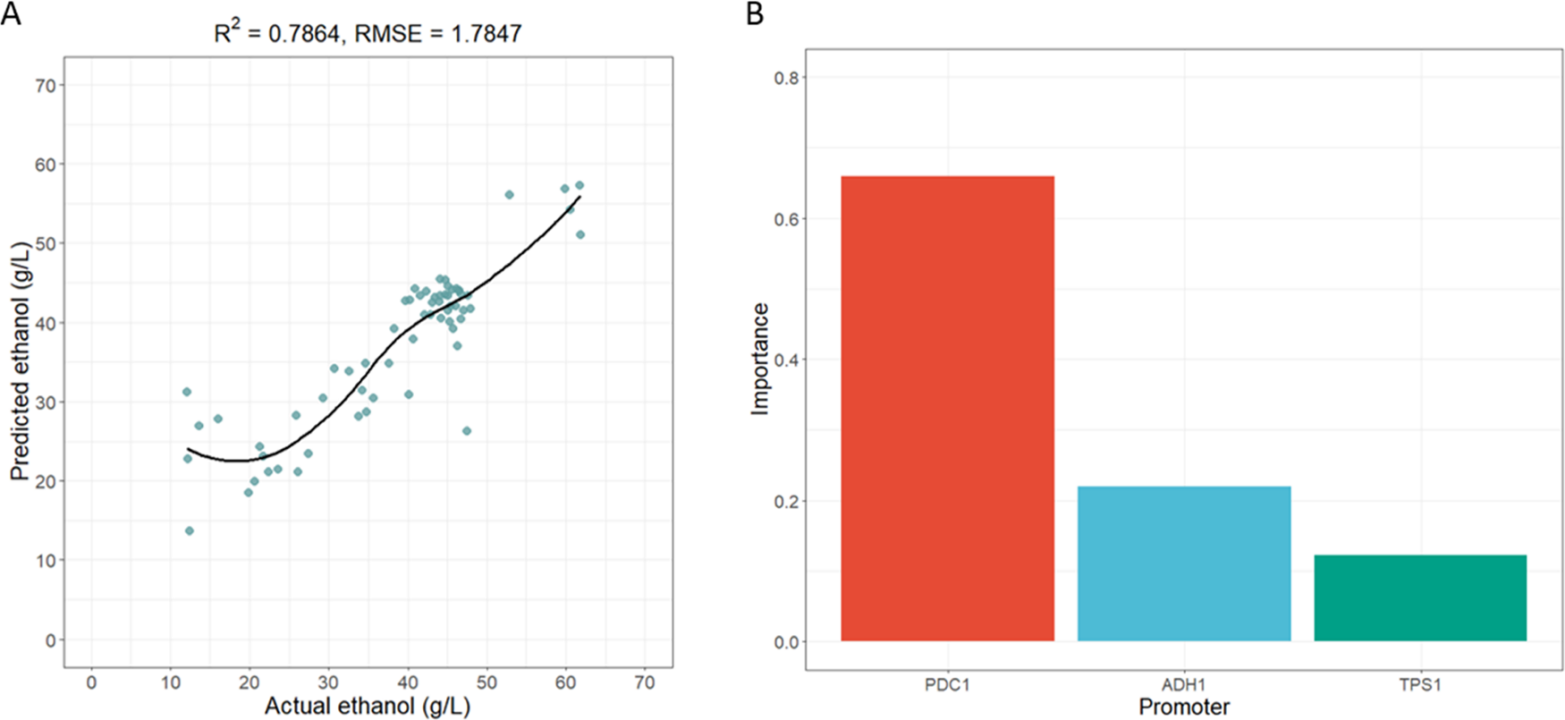
XGBoost tuning model reveals accurate prediction and low error. (A) The fine-tuned XGBoost model, using a random search strategy and hyperparameter tuning, exhibited improved *R*-squared values and decreased RMSE (root-mean-square error) in predicting ethanol production at 30 °C. (B) The distribution of important features from the fine-tuned XGBoost model indicated that the promoter of *PDC1* was the most important in the model, followed by the promoters of *ADH1* and *TPS1*.

For XGBoost training, we used promoter strengths and ethanol production data from 152 strains (70%) as input to train the model. First, we applied a random search strategy to tune the hyperparameters, using 1000 combinations of the learning rate, maximum depth of tree, minimum sum of instance weight, subsample ratio of training instance, and subsample ratio of columns. We then used the tuned hyperparameters to find the best-boosting iteration of the model using 5-fold cross-validation. Compared to the default training, the RMSE of training decreased from 7.70 to 1.78, and the *R*-square from test data (30%, 64 strains) prediction increased from 0.69 to 0.78 (Figure 4A). Therefore, tuning the XGBoost model with hyperparameters and cross-validation demonstrated an improved performance of the training model and prevented overfitting. These results indicate that XGBoost was suitable for training promoter strength combinations and predicting metabolite outcomes, and the variability of model performances can be found in the Supporting File 7. We then used the same workflow to predict the levels of intermediate metabolites, such as pyruvate, acetate, etc. Predicting pyruvate output showed an even higher *R*-square (0.83) and relatively low error (0.05), whereas predicted performances for acetate and glycerol were lower (Table 1).

**Table 1:** ML Model Performances from Fine-Tuning XGboost Model Using Levels of Ethanol and Intermediate Metabolites, OD_600_, and % Glucose Consumption as Individual Predicted Output

output	train RMSE	*R*-square	best iterations	eta	max.depth	subsample	colsample by tree	min child weight
EtOH (g/L)	1.7847	0.7864	42	0.1	9	1	1	0
EtOH (g/L/OD_600_)	0.073	0.7187	8	0.5	9	0.8	1	1
OD_600_	0.7145	0.7756	15	0.3	10	0.9	1	1
pyruvate	0.0523	0.829	11	0.4	6	0.9	1	1
acetate	0.126	0.7182	20	0.2	9	1	0.9	1
glycerol	0.2164	0.61	13	0.4	5	1	0.9	1
%glucose consumption	8.4636	0.7502	43	0.1	7	1	0.9	2

In contrast to deep learning, the XGBoost model provides some insight into the contribution or importance of each parameter in making predictions. To investigate which gene’s expression (i.e., *PDC1*, *ADH1*, or *TPS1*) has the most significant influence on ethanol prediction, we obtained the feature importance of different training models using various predicted metabolites. The promoter strength of *PDC1* was the most important for predicting ethanol production (65.87%), while the promoter of *ADH1* contributed 21.91%. Although *TPS1* was not directly involved in the ethanol production pathway, the promoter strength of this gene also contributed 12.20% (Figure 4B and Supporting Table 2). This ruling decision on how the training model predicted the metabolite levels was consistent with the earlier result that *pTDH3* at the *PDC1* gene exhibited high ethanol production. Furthermore, the contribution of the *PDC1* promoter was more significant than that of the *ADH1* and *TPS1* promoters in other models. This suggests that the levels of pyruvate, acetate, and glycerol are more affected by *PDC1* than those of the other two genes. *PDC1* encodes the enzyme pyruvate decarboxylase, which converts pyruvate to acetaldehyde. Acetaldehyde is then converted to acetate by the enzyme acetaldehyde dehydrogenase.^9,39^ Changes in *PDC1* transcription may predominantly affect the metabolic flux of the pyruvate and ethanol production pathway intermediates. When the contribution of the *ADH1* promoter was compared with all predicted outputs, it was found to be the most significant predictor of glucose consumption. Glucose consumption is directly related to growth and ethanol production because the amount of glucose used indicates how well the yeast grows and acts as a primary substrate for ethanol production.

Although the input data for machine learning in this study was limited by the combination of genes and promoter strengths, our XGBoost workflow demonstrated that the amount of input used in this case was sufficient to train the model.^26,28^ XGBoost regression has been used to predict promoter strength from the synthetic promoter library^40^ and genotype–phenotype biosensors,^41^ demonstrating the algorithm’s competence for other predictions in metabolic engineering. Furthermore, incorporating other related factors, including intermediate metabolites and growth parameters, may facilitate the prediction and selection of optimized yeast. This workflow for strain optimization may reduce the cost and laboriousness of experimental iteration and accelerate the optimization process in the DBTL cycle.^26,30,32^

### Validation of XGBoost Workflow in Predicting Ethanol Production under Heat Stress

Encouraged by the performance of the XGBoost workflow we developed in predicting ethanol production at an optimal growth temperature of 30 °C, we next set out to validate whether the workflow could also be used to optimize ethanol production at an elevated temperature of 40 °C. We performed ethanol fermentation at 40 °C using a selected set of 31 combinatorial strains out of the total 216 strains. These 31 strains were chosen to contain varying promoter strength combinations of *PDC1*, *ADH1*, and *TPS1* genes. We also ensured that the selected strains produced ethanol at diverse titers to closely represent the entire combinatorial library using earlier ethanol production from a temperature of 30 °C.

**Figure 5:**
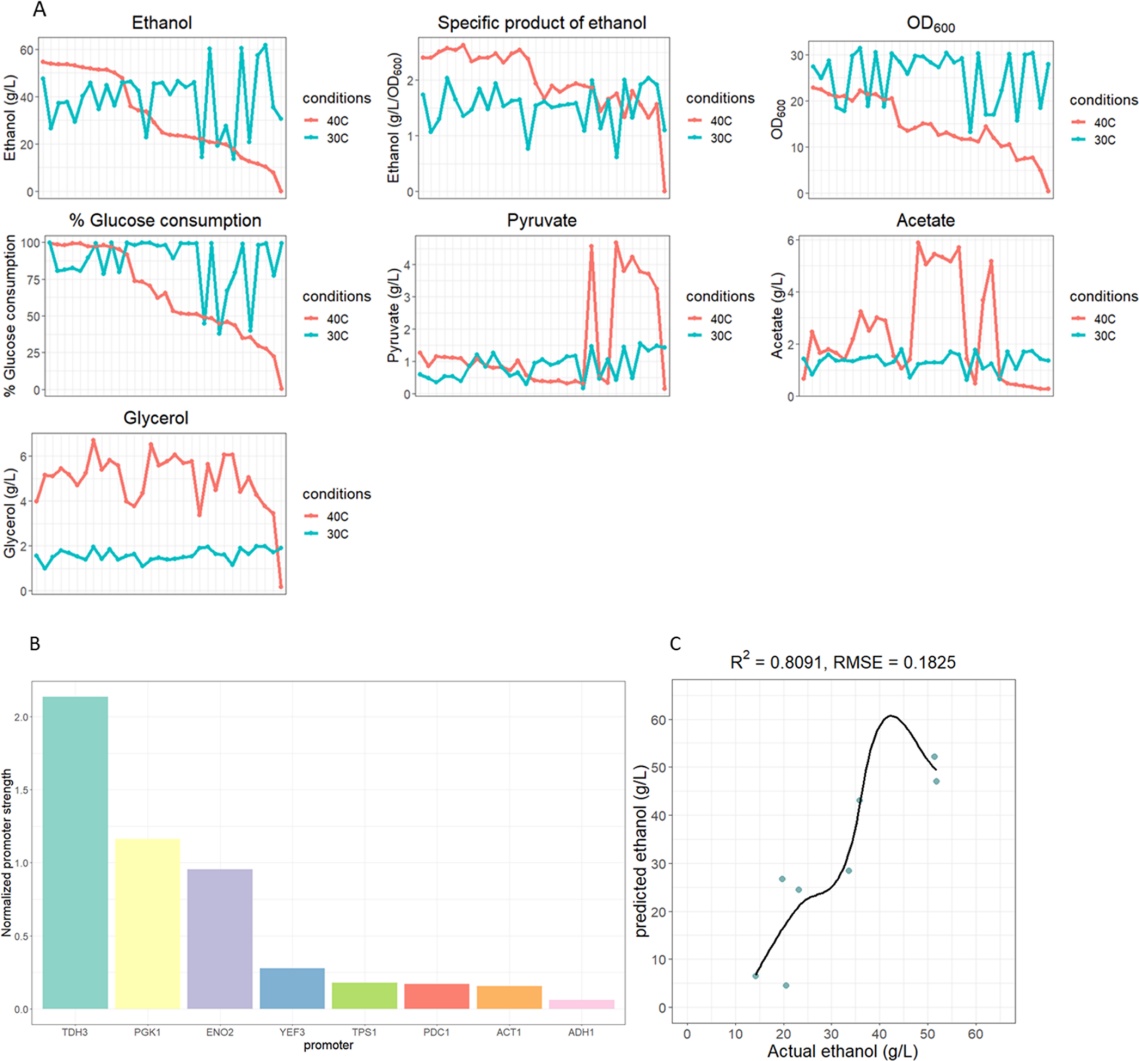
Improvement of ethanol production at 40 °C using the XGBoost workflow. (A) Comparison of levels of ethanol and intermediate metabolites, OD_600_, and % glucose consumption between 30 and 40 °C ethanol production using selected combinatorial strains. The *x*-axis represents 31 ordered combinatorial strains from high to low ethanol production, including WT. Productions at 30 °C (blue line) were plotted against those at 40 °C (pink line). (B) GFP intensities at 40 °C were normalized by OD_600_ and then scaled to use as input for ML training of the 40 °C ethanol production. (C) Fine-tuning the XGBoost model using a random search strategy and hyperparameter tuning exhibited high *R*-square values and low RMSE from training data (23 strains) and test data (8 strains).

Under heat stress conditions, three strains from our selection of 31, specifically NxN, KxK, and xKN, produced ethanol at levels comparable to those of the wild-type strain (53.58 g/L). While their ethanol production levels were among the highest in the selected strains, they did not significantly exceed the ethanol levels of the wild-type strain, recording 54.78 ± 0.45, 54.84 ± 0.77, and 53.59 ± 1.39 g/L for NxN, KxK, and xKN, respectively. Notably, one shared feature between these three strains was the *pENO2* promoter driving the *TPS1* gene. These high ethanol production strains were also associated with high growth (OD_600_), glucose consumption, and specific ethanol production. On the other hand, their pyruvate levels were relatively low compared to the levels observed in other strains. There appears to be no correlation between the levels of acetate and glycerol in high ethanol production strains (Figure 5A and Supporting Figure 2).

Next, we compared the changes in ethanol production under normal and heat stress conditions. Interestingly, we found no correlation between ethanol production at normal and high temperatures. For instance, strain TAA had the highest ethanol production at 30 °C but produced 10.24 ± 2.4 g/L of ethanol at 40 °C. This suggests that heat stress significantly affects ethanol production. Fine-tuning of gene expression in combinatorial strains to improve ethanol production at 30 °C differed under heat stress conditions. The drastic differences in the strains’ ethanol production at the two temperatures prompted us to measure promoter activity at 40 °C. We found that the activities of *pTDH3*, *pPGK1*, and *pENO2* remained the strongest, while the strengths of *pPDC1* and *pACT1* decreased relative to *pTPS1*’s level under heat stress conditions (Figure 5B).

To train the XGBoost model using promoter strengths and metabolites at 40 °C, we divided the 31 data points (one for each strain) into 70% training data (23 strains) and 30% test data (8 strains). We used the random search strategy to tune hyperparameters, as in the 30 °C model, and 5-fold cross-validation to prevent overfitting. The tuned XGBoost model exhibited an RMSE of 0.18 and an *R*-square of 0.81 from the test data predictions (Figure 5C). We also trained the XGBoost model using other metabolites as predicted outputs. The results are shown in Supporting Table 3. OD_600_ and glucose consumption showed higher R-squared values of 0.74 and 0.90, respectively, compared to those of other factors. Therefore, we used the three tuned XGBoost models (ethanol production, OD_600_, and glucose consumption) to predict which of the remaining combinatorial strains would produce the largest amount of ethanol.

The top 30 combinatorial strains from each prediction model of ethanol, OD_600_, and glucose consumption were intersected, resulting in the top 10 strains for experimental validation (Supporting Figure 3). From the 10 strains selected for experimental validation, Nxx produced the highest amount of ethanol, at 55.99 ± 0.12 g/L, which is higher than those of all 30 selected strains from the first round of experiments. Moreover, three more combinatorial strains, including KxY, Nxx, and xKY, showed higher ethanol production compared to the wild type (53.58 ± 1.07 g/L) (Supporting File 3). This demonstrates that the XGBoost workflow for strain optimization by modifying the promoter strengths of key pathway genes can reduce laborious work and accelerate optimization. In the optimization of ethanol production at 40 °C, we performed experiments using a total of 41 strains, which was approximately one-fifth of all 216 combinatorial strains. Furthermore, this approach can also suggest which genes are necessary for optimization. However, the small amount of input data for training can affect prediction accuracy, as some of our predicted strains produced low ethanol concentrations, such as in the KYN strain.

We incorporated data from the 10 predicted strains into the model to train it and predict new potential strains. In the second round of learning, the model predicted several strains with higher ethanol production. The optimized strains showed increased promoter strength for TPS1, such as xxN, xxA, xNA, and xKA, and the ethanol titer was increased up to 7.4% (57.55 ± 1.13 g/L) under the heat stress condition (see Supporting Table 4 and Supporting File 4).

Our approach parallels the iterative optimization processes used in studies such as the production of β-carotene and violacein, which incorporated machine learning algorithms for optimization. Notably, in these studies, substantial production improvements were achieved in the second round of β-carotene optimization.^31^ Similar iterative, ML-guided metabolic optimization strategies have been successfully applied in the optimization of protein production,^28^ monoterpenoid production,^26^ and tryptophan productivity.^32^ The top candidates from the prediction were selected for experimental validation and improved with iterated rounds. Although the results were promising, our study also highlighted the challenges and limitations of the current approach. Heat stress induces complex adaptations in yeast,^18^ and merely introducing changes in *TPS1* expression proved insufficient to significantly enhance ethanol production. This limitation could be resolved by increasing the number of promoters and the number of modified genes involved in heat stress tolerance. Moreover, this prediction involves two traits, ethanol production and heat stress tolerance, that may be interconnected through a complex mechanism. To minimize errors in future experiments, we suggest using heat-tolerant strains as the starting background for optimizing ethanol production using the XGBoost workflow or vice versa.

## Conclusions

In summary, we have demonstrated a machine learning workflow to assist in strain optimization. Using the CRISPR–Cas9 system, we varied the promoter strengths of genes encoding rate-limiting enzymes in ethanol production, including *PDC1* and *ADH1*, as well as the heat stress response gene *TPS1* involved in trehalose synthesis. Subsequently, we generated 216 fine-tuned combinatorial yeast strains showing an increase of approx. 63% of ethanol production. We used XGBoost to train the model with promoter strengths and ethanol production as the input. We have shown that fine-tuning the hyperparameters using the random search strategy and 5-fold cross-validation improved the training error and predictive accuracy. This revealed the successful establishment of the ML-to-ethanol optimization workflow. We then performed ethanol production at an elevated temperature to experimentally validate this approach. The second round of test and learning cycles exhibited improvement in ethanol production from predicted candidates up to 7.4%, although there was a limitation on the amount of training data.

Overall, our workflow successfully predicted promoter strength combinations that led to enhanced ethanol production under optimal growth conditions as well as under heat stress, demonstrating its applicability under challenging environmental conditions. This workflow has the potential to reduce laborious experimental iterations and accelerate the optimization process in the DBTL cycle. Our findings contribute to the ongoing efforts in metabolic engineering and pave the way for the development of robust microbial strains for industrial-scale biofuel production under varying environmental conditions.

## Materials and Methods

### Strain Constructions, Media, and Yeast Transformation

The yeast strains used in this study were constructed from *S. cerevisiae* CEN.PK2–1C, a laboratory strain with the genotypes *MAT*a; *his3Δ1*; *leu2–3,112*; *ura3–52*; *trp1–289*; *MAL2–8c*; and *SUC2*. The CRISPR–Cas9 plasmids used for genome editing were generated from p414–TEF1p–Cas9–CYC1t and pRPR1–gRNA handle,^42,43^ and the primers used are listed in Supporting File 5. The GFPuv plasmids used for quantifying promoter strengths were constructed from p416Tef1–URA3.^44^ Yeast transformation was performed using the LiAc/SS carrier DNA/poly(ethylene glycol) (DNA/PEG) method as previously described.^45,46^ The transformants were selected on yeast minimal medium, which contained 6.7 g/L of yeast nitrogen base with ammonium sulfate without amino acids (Difco), 2% of glucose, and a mixture of appropriate nucleotide bases and amino acids with dropouts. To evaluate ethanol production at various temperatures, yeast strains were cultivated in 5 mL of YPD10 medium, which contained 2% peptone, 1% yeast extract, and 10% glucose, in a 50 mL Falcon tube with 250 rpm of agitation for 24 h.

### Plasmid Constructions and Donor DNA Amplification

To construct pRPR1–gRNA–PDC1pro, pRPR1–gRNA–ADH1pro, and pRPR1–gRNA–TPS1pro, which are guided RNA plasmids for promoter replacement, the promoter-specific fragments were amplified from pRPR1–gRNA handle–RPR1t using forward primers PDC1pro_gRNA_F, ADH1pro_gRNA_F, or TPS1pro_gRNA_F and the reverse primer gRNA_Rev (Supporting File 5). The crRNA sequences were designed by the CRISPR RGEN tool.^47^ The fragment was then gel-purified and ligated to the *Hin*dIII/*Xho*I site of the pRPR1–gRNA handle–RPR1t. The DNA sequence was verified by DNA sequencing.

For the construction of the GFP reporter plasmid, the *GFPuv* gene was amplified from pGFPuv using primers GFPuv_*Bgl*II_F and GFPuv_ *Eco*RI_R. The fragment was then gel-purified and ligated to the *Bam*HI/*Eco*RI site of p416Tef1–URA3 to create p416Tef1–GFPuv. The five endogenous promoters of *ACT1*, *PGK1*, *ENO2*, *TDH3*, and *YEF3* genes were amplified from *S. cerevisiae* genomic DNA using the primers listed in Supporting File 5. The purified fragments were cloned into the *Spe*I site of p416Tef1–GFPuv to create p416ACT1–GFPuv, p416PGK1–GFPuv, p416ENO2–GFPuv, p416TDH3–GFPuv, and p416YEF3–GFPuv, respectively.

The promoter donor DNA was amplified using the genomic DNA of the *S. cerevisiae* CEN.PK2–1C strain as a template and Phusion DNA polymerase (Thermo Scientific). The primers used for donor DNA amplification are listed in Supporting File 5. To replace the target promoter through homologous recombination, the upstream region of the target promoter and the start of the coding sequence were added to the 5′ and 3′ ends of each donor DNA, respectively.

### Determination of Promoter Strength

Yeast strains harboring the individual GFP-reporting plasmids were inoculated in 5 mL of synthetic complete media (10% glucose) without uracil and incubated at temperatures of 30 and 40 °C with 250 rpm of agitation for 24 h. The cells were harvested and washed twice with sterile distilled water. They were then resuspended in water at a dilution of 10:1. Green fluorescence was excited at 395 nm, and the emission at 509 nm was measured using the SynergyH1 microplate reader (Biotek, Agilent). The fluorescence intensity was normalized by cell density, and the average was taken from triplicate.

### Small-Scale Ethanol Production

To evaluate ethanol production at 30 °C, a single colony of each yeast strain was inoculated into 5 mL of YPD10 medium and incubated with 250 rpm shaking for 24 h. For production at 40 °C, a preculture of the yeast strain was prepared in 5 mL of YPD at 30 °C with 250 rpm agitation for 18 h. The cells were then harvested at 3000*g* for 5 min, resuspended in YPD10, and inoculated into 5 mL of YPD10 to achieve an optical density at 600 nm (OD_600_) of 0.25. The yeast cells were then incubated at 40 °C for 24 h with 250 rpm agitation.

The yeast culture supernatant was filtered through a 0.2 μm nylon syringe filter (Filtrex, Thailand). The filtered sample was analyzed using an Agilent 1100 series HPLC with the Shodex sugar SH1011 column (8.0 mm ID × 300 mm) to quantify ethanol, glycerol, acetate, and pyruvate production. Glucose consumption was calculated from the remaining glucose. The mobile phase was 5 mM H_2_SO_4_, with a flow rate of 0.5 mL/min at 70 °C for 40 min. Ethanol production was performed using all 216 combinatorial strains with three biological replicates.

### Machine Learning Model Training and Tuning

#### Machine Learning Algorithm Exploration

The promoter strengths from the GFP intensity measurement were normalized by OD_600_ and then scaled. Promoters of *PDC1*, *ADH1*, and *TPS1* were replaced by five endogenous promoters to create all 216 possible combinations, including the native promoter of each gene (6 *PDC1* × 6 *ADH1* x 6 *TPS1* = 216). Each combination of normalized promoter strengths from *PDC1*, *ADH1*, and *TPS1* and ethanol production at 30 °C was used as input for exploring machine learning algorithms. All machine learning methods employed in this study were optimized according to available hyperparameters in the caret package.^33^ We used the caret package^33^ to examine suitable machine learning algorithms, including linear regression (by lm), generalized linear regression (by glmnet^48^), decision trees (by rpart^49^), random forest (by rf^50^), extreme gradient boosting (by xgboost^51^), and support vector machine (by kernlab^52^). The training and test data sets were divided into 70 and 30% of the total 216 combinatorial strains, respectively. All training models were optimized according to each available hyperparameter in the caret package. In the generalized linear model constructed using the glmnet library, we tuned the α and lambda parameters during model training. For decision tree and random forest constructed with rpart and randomForest libraries, respectively, we optimized the cp and mtry parameters. For the XGBoost model, we optimized hyperparameters including eta, max_depth, γ, colsample, min_child_weight, subsample, and nrounds. Finally, for the support vector machine, we optimized the scale, *C*, and degree parameters.

The performance of the training model and prediction accuracy were calculated by using mean absolute error (MAE), root-mean-square error (RMSE), and *R*-square (*R*^2^). Training and test data were randomly split 20 times to determine the performance variability

#### Training and Tuning of the XGBoost Model

A scalable and flexible gradient tree boosting algorithm called XGBoost^37^ was applied for predicting ethanol production using combinations of modified promoter strengths as input, together with concentrations of ethanol and other metabolites. Data from 216 combinatorial strains in the 30 °C fermentation experiment were randomly separated into 70% training sets (152 combinatorial strains) and 30% test sets (64 combinations). To train and tune the XGBoost model, we first used a random search strategy^53^ that created 1000 combinations of hyperparameters, including maximum depth of trees, eta, minimum sum of instance weight, subsample ratio of the training instances, and subsampling of columns. This hyperparameter tuning facilitated the model’s performance and prevented overfitting, in parallel with 5-fold cross-validation. Finally, the performance of the fine-tuned XGBoost model was evaluated by using the RMSE of the training model and prediction accuracy (*R*^2^). In addition, we applied the same training workflow to predict ethanol production at 40 °C. Training and testing data contained 23 and 8 combinations, respectively, from all 31 selected combinatorial strains. Training and test data were randomly split 30 times to determine the performance variability in both models.

All machine learning analyses were conducted using R version 4.1.1^54^ with the XGboost^51^ and caret packages.^33^ Visualized plots were generated using the ggplot2 package.^55^ Source codes with detailed parameters are available on the GitHub repository, https://github.com/IMCTlab/ML-ethanol-optimization. Raw and processed data are included in the repository and Supporting Files.

## Supporting Information Available

Combinatorial strain annotations from Figure 2A–F (Figure S1); comparison of ethanol production and intermediate metabolites at 40 °C (Figure S2); overlap of top 30 strain candidates from individual model predictions of ethanol production, OD_600_, and % glucose consumption (Figure S3); machine learning performances (Table S1); feature importance from training models (Table S2); ML model performances from fine-tuning XGboost model from the 40 °C candidates (Table S3); and hyperparameters for the second round tuning of the XGBoost model (Table S4) (PDF)
Average and STD data from 30 °C ethanol and metabolite productions (Supporting File 1) (XLSX)
Predicted and measured ethanol levels at 30 °C (Supporting File 2) (XLSX)
Predicted and measured ethanol and other metabolite production at 40 °C (Supporting File 3) (XLSX)
Second round ML predicted and measured ethanol levels using the tuned 40 °C EtOH XGBoost model (Supporting File 4) (XLSX)
Primers used in this study (Supporting File 5) (XLSX)
20train-test splits 5ML methods (Supporting File 6) (XLSX)
30train-test splits XGBoostoptmization 30 and40 °C (Supporting File 7) (XLSX)
