# Supplementary material for: Optimizing Ethanol Production in Saccharomyces cerevisiae at Ambient and Elevated Temperatures through Machine Learning-Guided Combinatorial Promoter Modifications
Source: ACS Synth Biol. 2023 Sep 8;12(10):2897–908. doi: 10.1021/acssynbio.3c00199 (PMC10594650; doi:10.1021/acssynbio.3c00199)
Supplement: Supplementary file 1 — sb3c00199_si_001.pdf [file sb3c00199_si_001.pdf]

Supporting Figures and Tables for Optimizing Ethanol Production in *Saccharomyces cerevisiae* at Ambient and Elevated Temperatures through Machine Learning-Guided Combinatorial Promoter Modifications

Peerapat Khamwachirapithak[1,#], Kittapong Sae-Tang[1,#], Wuttichai Mhuantong[1], Sutipa Tanapongpipat[1], Xin-Qing Zhao[2], Chen-Guang Liu[2], Verawat Champreda[1], and Weerawat Runguphan[1,*]

[1] National Center for Genetic Engineering and Biotechnology (BIOTEC), National Science and Technology Development Agency (NSTDA) 111 Thailand Science Park, Phahonyothin Road, Khlong Nueng, Khlong Luang, Pathum Thani, 12120, Thailand

[2] State Key Laboratory of Microbial Metabolism, Joint International Research Laboratory of Metabolic & Developmental Sciences, School of Life Sciences and Biotechnology, Shanghai Jiao Tong University, Shanghai, 200240, China

[#] These authors contributed equally to this work.

[*] Correspondence: weerawat.run@biotec.or.th

Supporting Figure 1.
Combinatorial strain annotations from Figure 2A-2F

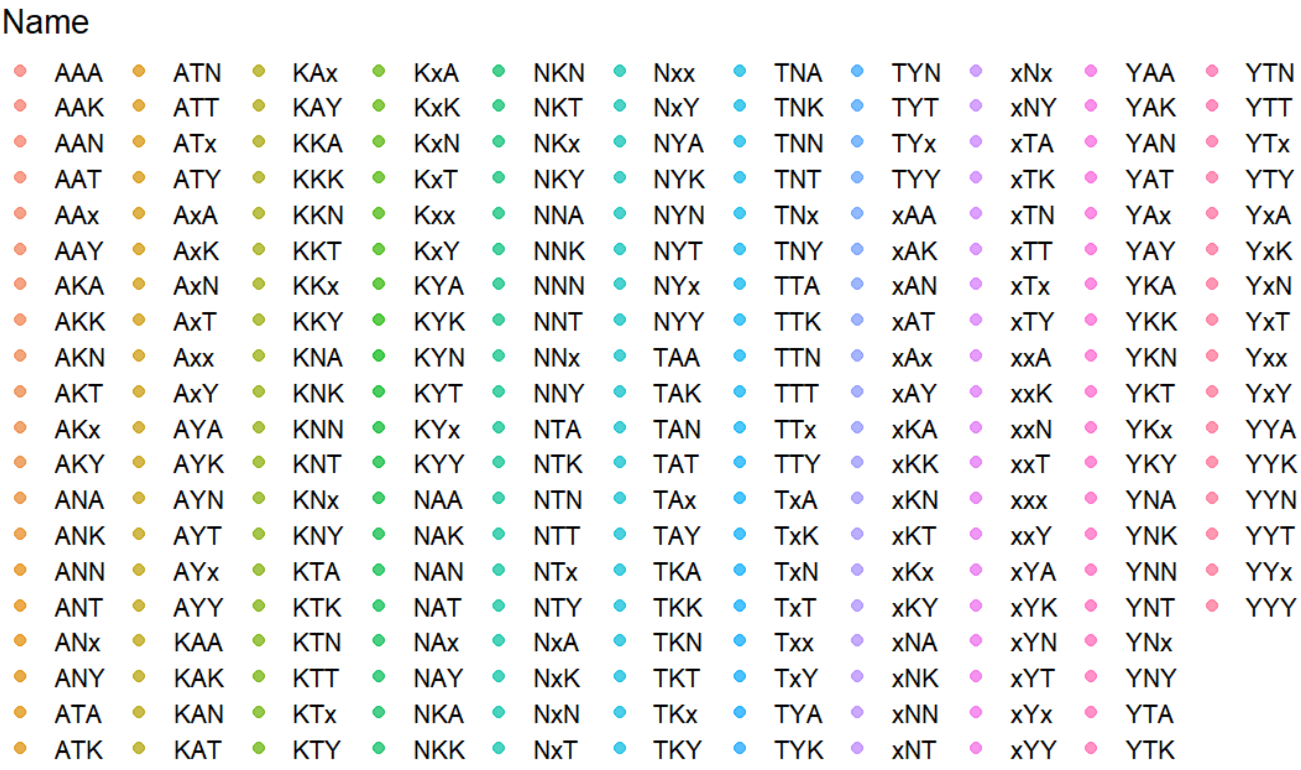

Supporting Figure 2.
Comparison of ethanol production and intermediate metabolites at 40 °C

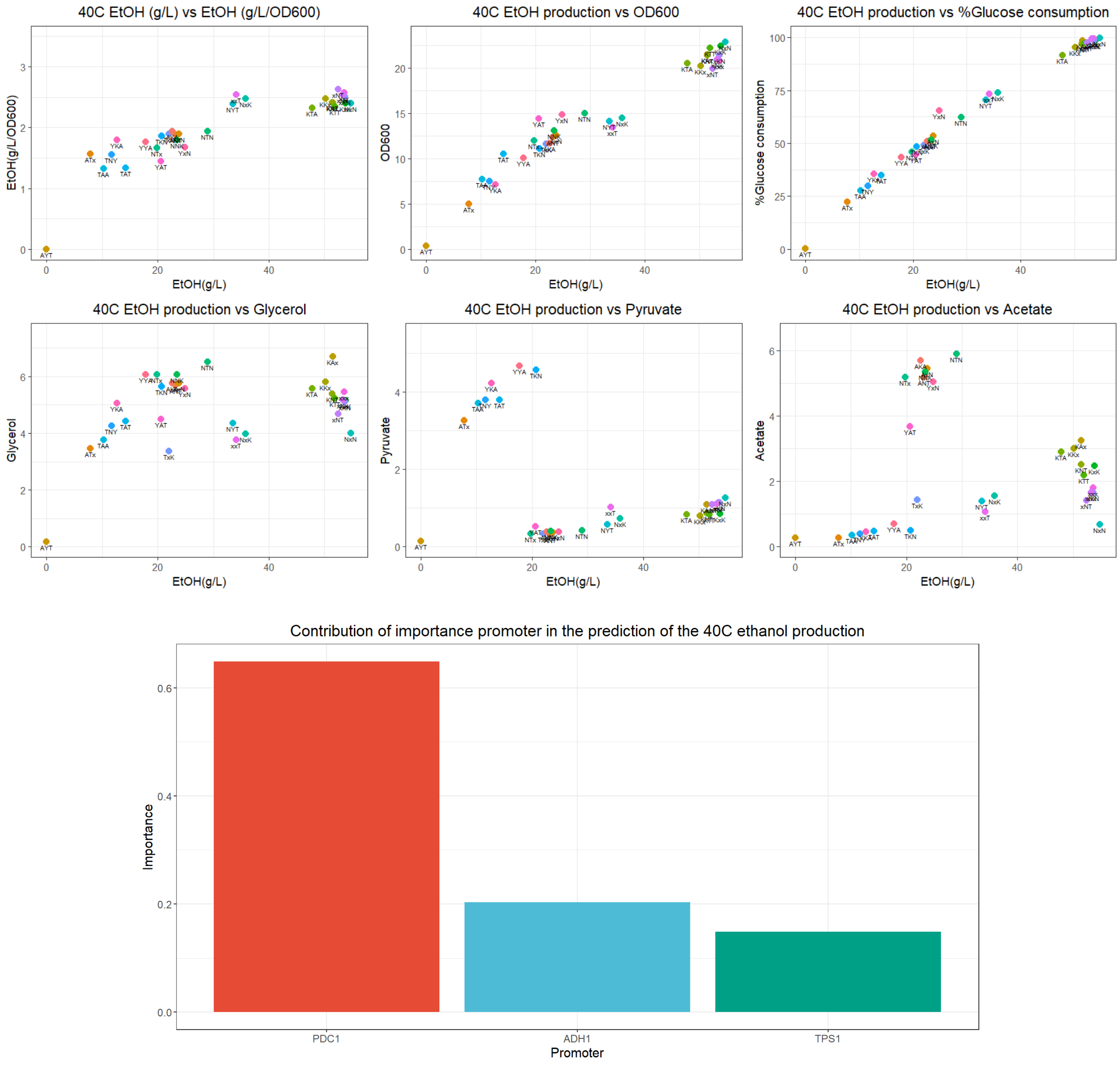

Supporting Figure 3. Overlap of top 30 strain candidates from individual model predictions of ethanol production, OD_{600}, and % glucose consumption.

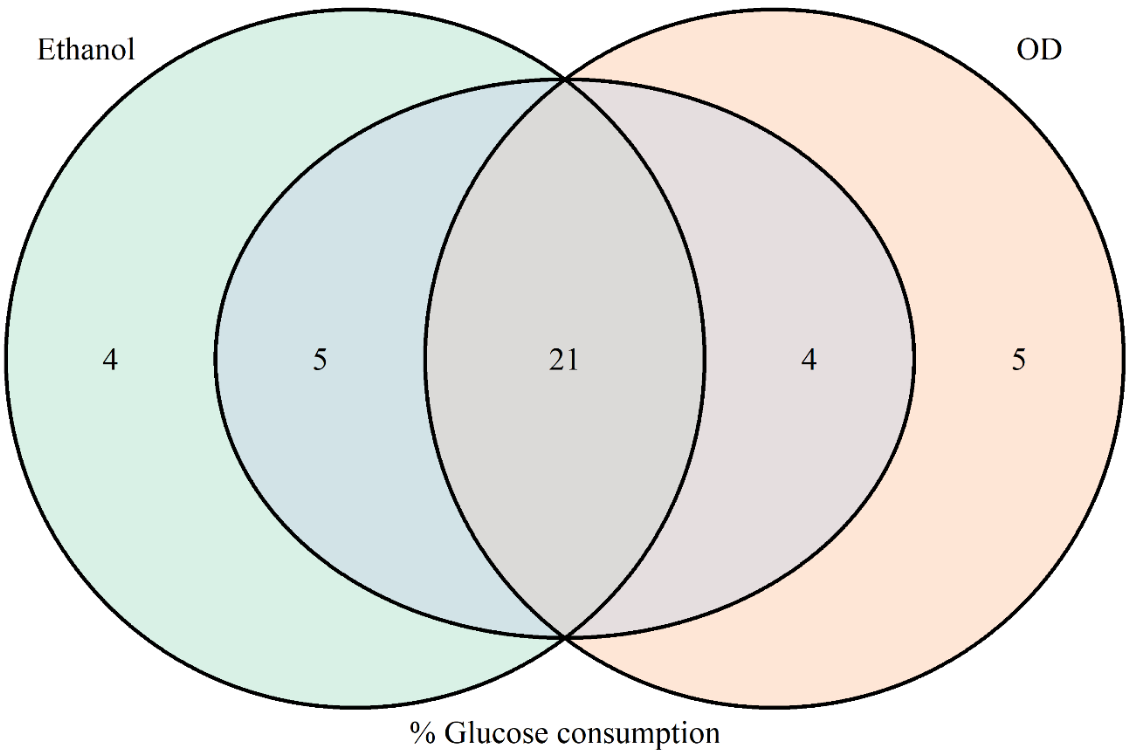

Supporting Table 1. Machine learning performances

Model	Train			Test		
	R^2	MAE	RMSE	R^2	MAE	RMSE
Linear regression	0.0204	8.98	11.318	0.0122	9.6578	12.1076
Generalized Linear Model	0.1313	9.2388	11.6251	0.0141	9.6621	12.0924
Decision Tree	0.2680	6.7076	9.9719	0.3461	6.3376	9.7784
Random Forest	0.5957	5.3963	5.5957	0.7711	4.5795	6.2145
XGBoost	0.5674	5.716	7.6954	0.6852	5.1883	6.9188
Support Vector Machine	0.02633	8.3514	12.1514	0.0115	9.0012	12.1291

Supporting Table 2. Feature importance from training models

Output	PDC1	ADH1	TPS1
EtOH (g/L)	0.659	0.219	0.122
EtOH (g/L/OD_{600})	0.413	0.37	0.274
OD_{600}	0.549	0.238	0.213
Pyruvate (g/L)	0.497	0.336	0.167
Acetate (g/L)	0.517	0.378	0.105
Glycerol (g/L)	0.553	0.383	0.083
%Glucose consumption	0.493	0.416	0.091

Supporting Table 3. ML model performances from fine-tuning XGboost model from the 40 °C candidates

Output	Train RMSE	R-square (Predicted vs measured)	Best iterations	eta	Max.depth	subsample	Colsample by tree	Min child weight
EtOH (g/L)	0.1824	0.809	61	0.5	3	1	0.7	0
EtOH (g/L/OD_{600})	0.0706	0.4524	33	0.2	4	0.9	0.9	1
OD_{600}	0.1978	0.7376	44	0.3	4	1	0.7	0
Pyruvate (g/L)	0.2924	0.4311	10	0.4	7	0.9	0.7	1
Acetate (g/L)	1.6712	0.1823	3	0.3	8	0.8	0.8	1
Glycerol (g/L)	1.2543	0.4415	7	0.4	6	0.8	0.9	8
%Glucose consumption	0.269	0.9027	57	0.4	4	1	0.7	1

Supporting Table 4. Hyperparameters for second round tuning of the XGBoost model

Output	Train RMSE	R-square (Predicted vs measured)	Best iterations	eta	Max.depth	subsample	Colsample by tree	Min child weight
EtOH (g/L)	8.4454	0.6902	22	0.3	9	0.9	0.6	0
